# Eliciting and prioritising determinants of improved care in multimorbidity: A modified online Delphi study

**DOI:** 10.1177/26335565231194552

**Published:** 2023-09-05

**Authors:** Glenn Simpson, Beth Stuart, Marisza Hijryana, Ralph Kwame Akyea, Jonathan Stokes, Jon Gibson, Karen Jones, Leanne Morrison, Miriam Santer, Michael Boniface, Zlatko Zlatev, Andrew Farmer, Hajira Dambha-Miller

**Affiliations:** 1 Primary Care Research Centre, 12211 University of Southampton, Southampton, UK; 2 Wolfson Institute of Population Health, 105714 Queen Mary University of London, London, UK; 3 Institute of Epidemiology and Health Care, 4919 University College London (UCL), London, UK; 4 MRC/CSO Social and Public Health Sciences Unit, 3526 University of Glasgow, Glasgow, UK; 5 Division of Population Health, Health Services Research and Primary Care, 5292 University of Manchester, Manchester, UK; 6 Personal Social Services Research Unit, 2240 University of Kent, Canterbury, UK; 7 School of Psychology, 12211 University of Southampton, Southampton, UK; 8 Digital Health and Biomedical Engineering, School of Electronics and Computer Science, 7423 University of Southampton, Southampton, UK; 9 Nuffield Department of Primary Care Health Sciences, 6396 University of Oxford, Oxford, UK

**Keywords:** Multimorbidity, Delphi study, integrated care, personalised care

## Abstract

**Background:**

Multimorbidity is a major challenge to health and social care systems around the world. There is limited research exploring the wider contextual determinants that are important to improving care for this cohort. In this study, we aimed to elicit and prioritise determinants of improved care in people with multiple conditions.

**Methods:**

A three-round online Delphi study was conducted in England with health and social care professionals, data scientists, researchers, people living with multimorbidity and their carers.

**Results:**

Our findings suggest a care system which is still predominantly single condition focused. ‘Person-centred and holistic care’ and ‘coordinated and joined up care’, were highly rated determinants in relation to improved care for multimorbidity. We further identified a range of non-medical determinants that are important to providing holistic care for this cohort.

**Conclusions:**

Further progress towards a holistic and patient-centred model is needed to ensure that care more effectively addresses the complex range of medical and non-medical needs of people living with multimorbidity. This requires a move from a single condition focused biomedical model to a person-based biopsychosocial approach, which has yet to be achieved.

## Background

Multimorbidity occurs when an individual has two or more co-existing long-term health conditions.^1^ The prevalence of multimorbidity is an immediate priority and a long-term challenge to health and social care systems around the world owing to the projected rise in the number of people aged over 65 years with these conditions.^1–3^ This is not only the case in high-income countries where multimorbidity is “considered the norm, not the exception”^4^, but prevalence is also increasing rapidly in lower-income states. Globally, this will impose significant challenges at individual, care system and wider societal levels, as multimorbidity requires more intensive and complex care and is associated with higher levels of service usage and expenditure, increased sickness absence rates and a greater risk of social vulnerability (including homelessness, unemployment and poverty).^4–6^

Multimorbidity is also a challenge for professionals providing care for this cohort, as health and social care systems (including clinical training and clinical practice guidelines),^7^ are primarily based on a single disease paradigm.^8,9^ This is problematic, as the existing focus on single disease approaches based on a highly specialised care and treatment regime,^8^ may not comprehensively address the complex range of care needs which are a feature of multimorbidity.^10^ A recent scoping review^10^ found evidence that experiences of living with and seeking care for multimorbidity are not only shaped by medical factors and the quality of clinical care, but also by wider non-medical determinants (also referred to as social determinants) of health^11^ such as housing conditions, income and employment, and the quality of the local environments (access to green space, air quality, and neighbourhood crime levels and social cohesion). These non-medical determinants interact with each other and also with medical factors to determine an individual’s probability of developing multimorbidity and their subsequent deterioration in health.^9–11^ Whilst there are potentially a limitless number of medical and non-medical determinants that could be examined across all dimensions of care, it is unclear which of these determinants should be prioritised to deliver effective interventions for multimorbidity.^10^

Currently, there is a limited body of research exploring the full range of determinants that are critical to improving care in multimorbidity.^9,12^ Given these limitations in the evidence base, we elicited opinions from health and social care professionals, data scientists, researchers, people living with multiple conditions and their carers, in order to prioritise wider determinants of improved care in multimorbidity, with a view to informing efforts to develop new models of care that might address the holistic needs of this patient cohort.^13^

## Method

### Theoretical framework

The Sustainable intEgrated care models for multi-morbidity: delivery, FInancing and performancE (SELFIE) framework was used to conceptually inform our research design, methods and interpretations.^14^ SELFIE was developed as a framework to support the design, development, assessment and delivery of programmes of integrated care for individuals with multimorbidity.^14^ Theoretically, the framework is constructed around the holistic care needs of the individual with multimorbidity as experienced within their immediate environment. In this context, the individual with multiple conditions considered centrally, with surrounding and radiating concepts grouped into six adapted World Health Organisation components “used to describe, understand, and compare different health systems” (i.e.: 1. Service delivery; 2. Leadership and governance; 3. Workforce; 4. Financing; 5. Technologies and medical products; 6. Information and research).^14^ Within each component, concepts are further divided into three spatial scales (i.e. micro, meso and macro) that identify and prioritise the levels at which interventions, policy action and resources can be most effectively delivered by care providers to manage the care needs of those with multimorbidity.

### Study design

The Delphi technique is an established formal “group facilitation”^15^ method to gather anonymous opinions from a panel of experts with the aim of achieving consensus^16,17^ and is often used in research to determine priorities.^18^ This method was chosen for its flexibility, allowing us to elicit and prioritise a broad range of views on wider determinants relevant to improved care in multimorbidity. The study was reported using the guidelines in the Recommendations for the Conducting and Reporting of Delphi Studies (CREDES).^19^

### Recruitment and sampling

No agreed figure is available in the literature for the optimal number of participants required to conduct a Delphi study. Vogel et al.^20^ suggest that a “minimum of 12 respondents is generally sufficient to enable consensus.” Using this figure as a benchmark and taking into account the potential for participant ‘drop-out’ over the duration of the three rounds of the study,^21^ we aimed to recruit at least 20 participants. Purposive sampling was conducted to ensure a diverse range of participants in terms of backgrounds and experiences. We recruited via online forums, social media and the websites of relevant service provider organisations such as local government adult social care and voluntary sector groups. We also used snowball sampling whereby respondents who had agreed to be involved in the study were asked to recommend other participants to join the Delphi panel.^22^ All participants provided written consent to participate in the study.

### Survey development

The study was informed by the research question, a literature review, public and patient involvement and input from the wider study team.^17^ The study was piloted before being deployed. Each round included data collection of socio-demographic characteristics.

Round 1 asked a single open question: What medical and non-medical factors are key to improving the care of people with multiple long term conditions (e.g. medical, social, environmental, economic or other factors)? In response, participants were asked to provide up to 20 items.

**Figure 1:**
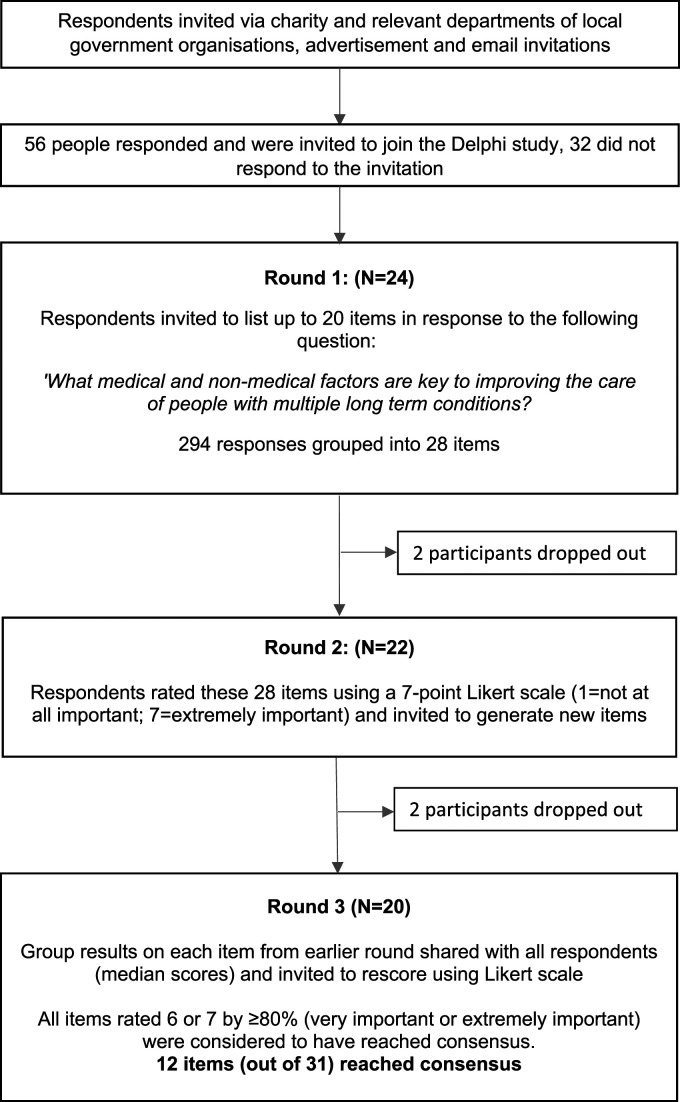
Flow chart summarising each stage of the Delphi process.

The purpose of round 1 was to encourage item generation by the participants with the subsequent rounds 2 and 3 focused on item prioritisation using a seven-point Likert Scale (ranging from: 1= not at all important, to 7= extremely important).^23^ Each round included a free-text box to allow respondents to add additional comments to elaborate further on their responses. Each stage of the Delphi process is set out in Figure 1.

### Data collection

Data collection was conducted between February and April 2022. A web-link to access the Delphi study was emailed to participants. An online format was used due to national restrictions imposed by the COVID-19 pandemic that limited in-person meetings and facilitated greater geographical diversity of participants. Those who expressed interest in the study were sent an online study information sheet. Non-responses were followed up with up to three email reminders sent to respondents. Following these reminders, participants were classified as ‘drop-outs’ if no response was received within a month.^24^ No participation incentives were used. The Delphi study lasted three rounds as sufficient consensus was achieved at this stage.

The data collected was transferred to the NVivo 12 qualitative analysis platform for collation and analysis. Consensus was defined by a priori criteria of 80% or more responses falling within the top two categories on the Likert Scale (6: very important or 7: extremely important).^25^

## Results

### Participants

All 56 respondents who expressed interest were invited to participate in the study. Of these 24 (43%) commenced round one and 20 of these initial respondents completed round three (83% follow-up). The flow of participants through each round is summarised in Figure 1.

An equal number of males and females completed the study, and the majority of respondents (83%) were from White British backgrounds. Most respondents resided in England (88%). All participants were over 18 years old. Those aged 40-49 were the single largest category of respondents (33%), although only one respondent was under 30 years old and three (13%) were in the oldest age range of 60-69 years, a period during the life course which is most commonly associated with multimorbidity prevalence. Respondents from care service providers and healthcare backgrounds were the two largest groups on the panel. A summary of the socio-demographic and occupational characteristics of the respondents (who initially participated in round 1) are shown in Table 1 below.

**Table 1.** Socio-demographic and occupational characteristics of respondents.

Respondent Characteristics	Results, n (%)
Gender	
Male	12 (50)
Female	12 (50)
Age	
18-29	1 (4)
30-39	6 (25)
40-49	8 (33)
50-59	6 (25)
60-69	3 (13)
Ethnicity	
British	20 (83)
Any other White background	2 (8)
Chinese	1 (4)
Mixed (White & Black Caribbean)	1 (4)
UK nation of residence	
England	21 (88)
Wales	1 (4)
Scotland	2 (8)
Current role/position	
Healthcare professionals	5 (21)
Social care professionals	3 (13)
Health data scientists	2 (8)
Researchers	3 (13)
Care service providers	6 (25)
Voluntary sector	3 (13)
Adults with multimorbidity	2 (8)

### Round 1

A total of 294 individual item responses were received in round 1. These items were grouped into related categories and labelled. The analysis was guided and structured using the SELFIE theoretical framework. Specifically, this involved the lead researcher systematically mapping items onto the SELFIE conceptual categories listed on Leijten et al’s^14^ diagrammatic model of integrated care for multimorbidity, a process repeated for additional items suggested in subsequent rounds (Table 2).

**Table 2.** Delphi determinants mapped onto SELFIE framework Those Delphi determinants highlighted in *italics* aligned with SELFIE framework.

Core of the framework	Levels of integrated care for multimorbidity			Six key components of a well-functioning health system
	Micro-scale	Meso-scale	Macro-scale	
Holistic understanding of the individual with multimorbidity:	Person-centred	Organisational and structural integration	Market regulation	Service Delivery
	Proactive	Continuous quality improvement system	Policies to integrate care across organisations and sectors	
Health				
Wellbeing	Tailored		Service availability and access	
Capabilities	Self-management			
	Informal caregiver involvement			
Self-management				
Needs				
	Treatment interaction			
Preferences				
	Continuity			
Environment:				
	Coordination tailored to complexity	Performance-based management	Policy and action plans on chronic diseases and multimorbidity	Leadership and Governance
Housing				
Social network				
Transport	Shared decision-making	Supportive leadership	Political commitment	
Community Welfare services Financing	Individualised care planning	Clear accountability		
		Performance-based management		
		Culture of shared vision, ambition, values		
			
	Multidisciplinary team	Continuous [professional] development	Educational and workforce planning	Workforce
	Named coordinator	Informal caregiver support	Workforce and demography match	

	Core Group	New professional roles		
			
	Coverage and reimbursement	Incentives to collaborate	Equity and access	Financing
	Out of pocket costs	Risk adjustments	Financial system for health and social care	
	Financial incentives	Shared savings	Stimulating investments in innovative care models	
		Secured budgets		
		Business case		
			
	EMR and patient portals	Shared information systems	Policies fostering technological innovation	Technologies and medical products
	E-health tools	Interoperable systems	Access to technologies and medical products	
	Assistive technologies			
	Remote monitoring			
			
	Individual data	Data ownership and protection	Access to information	Information and research
	Individual risk prediction	Innovative research [methods]	Policies that stimulate research in integrated care and multimorbidity	
		Risk stratification	Privacy and data protection legislation	

The mapping process identified items that did not clearly align with the SELFIE categories. The relevance of ‘non-aligned’ items and whether they should be included in subsequent rounds of the study were reviewed against the existing multimorbidity literature by the lead researcher. In addition, the findings of the mapping process were discussed with the wider 25-member study team, who included clinicians and experts in multimorbidity. These interactive discussions provided an additional layer of independent scrutiny over the conclusions reached about the relevance of individual items. Items not aligned with the SELFIE framework but considered relevant to the study’s aims were included in subsequent rounds for deliberation (these are marked in Table 3).

**Table 3.** Participant responses round 1.

Item Number	Suggested priority determinants of improved care in multimorbidity
1	Person-centred and holistic care
2	Continuity of care
3	Early intervention and preventative approach
4	Inclusive/Equitable service provision
5	Access to social care, community-based services and other provision
6	Service availability in terms of timeliness and geography
7	Training in multiple long term conditions for healthcare and social care professionals
8	Coordinated and joined up care
9	Data sharing across care services and linked electronic health records
10	Thorough assessment of health and social care needs
11	Supporting self-management of conditions
12	Symptom management and monitoring
13	Access to information for patients and carers and where to go for support
14	Use of technologies to support individuals at home
15	Prescribing and medication management
16	Enhanced support from family and other informal carers
17	Support provided to informal carers
18	Support network
19	Addressing loneliness and social isolation
20	Recognition of and support with lifestyle factors
21	Environmental factors and wider social determinant of health
22	Finance/financial assistance
23	Housing/accommodation that meets individual's needs
24	Increasing knowledge and awareness of multiple long term conditions among, patients, carers and people in the wider community ˜
25	Support for mental health and emotional wellbeing ˜
26	Able to engage in meaningful activities and social participation ˜
27	Support with daily living and independent living ˜
28	Work issues and training for people living with multiple long term conditions ˜

**˜** Items not aligned with the SELFIE Framework.

The mapping process synthesised the individual response items into 28 categories, defined as priority determinants (see Table 3). These priority determinants of improved multimorbidity were then presented to respondents in round 2.

### Round 2

Round 2 allowed respondents to rate the 28 determinants generated in round 1 (Table 4). Median scores were then calculated for each determinant based on the average of the Likert Scale rating. A clear hierarchy of prioritisation emerged, with the highest median scores given for systems-wide determinants ‘person-centred and holistic care’ (ranked 1) and ‘coordinated and joined up care’ (ranked 2). Determinants focused on care delivery, ‘thorough assessment of health and social care needs’ and ‘support for mental health and emotional wellbeing’, were ranked three and four respectively. The social determinant of health, ‘housing/accommodation that meets individual's needs’, also received a high rating, ranked five.

**Table 4.** Median ratings of priority determinants in round 2.

Number	Priority Determinants	Median
1	Person-centred and holistic care	7
2	Coordinated and joined up care	7
3	Thorough assessment of health and social care needs	7
4	Support for mental health and emotional wellbeing	7
5	Housing/accommodation that meets individual's needs	6.5
6	Continuity of care	6
7	Early intervention and preventative approach	6
8	Inclusive/Equitable service provision	6
9	Access to social care, community-based services and other provision	6
10	Service availability in terms of timeliness and geography	6
11	Training in multiple long term conditions for healthcare and social care professionals	6
12	Data sharing across care services and linked electronic health records	6
13	Supporting self-management of conditions	6
14	Symptom management and monitoring	6
15	Access to information for patients and carers and where to go for support	6
16	Prescribing and medication management	6
17	Addressing loneliness and social isolation	6
18	Support provided to informal carers	6
19	Environmental factors and wider social determinant of health	6
20	Finance/financial assistance	6
21	Able to engage in meaningful activities and social participation	6
22	Support with daily living and independent living	6
23	Increasing knowledge and awareness of multiple long term conditions among patients, carers and people in the wider community	5.5
24	Enhanced support from family and other informal carers	5.5
25	Recognition of and support with lifestyle factors	5.5
26	Work issues and training for people living with multiple long term conditions	5
27	Use of technologies to support individuals at home	5
28	Support network	5

Respondents were also able to suggest new determinants to add to the round 1 list. Three respondents proposed additional determinants for consideration in round 3: 1) ‘support for children of people living with multiple long term conditions’; 2) ‘more intervention from private care providers’; 3) ‘flexible systems in place to support people (before ‘cliff-edge’ moments when their conditions or circumstances change)’.

Three respondents added comments in the free text box, which were concerned with two themes: firstly, it was challenging to provide a rating, as the relative importance of each determinant depended on the personal circumstances of each individual and their specific care needs; and second, most determinants were interconnected, making it difficult to discriminate between their relative importance.

### Round 3

Respondents ‘re-prioritised’ the 28 determinants from earlier rounds after considering the group responses and also the three additional suggestions generated from the free-text responses in round 2. Respondents rated 12 out of 31 determinants as very important (6 or 7 on the Likert scale), thus reaching consensus as set out in our methods (Table 5).

**Table 5.** Final consensus results from round 3.

No.	Priority Determinants	Median score	Numbers (%) of participants who rated 6 (very important) or 7 (extremely important) N=20	Consensus level and priority rating of factors for addressing care need in multimorbidity	Ranking position
1.	Person-centred and holistic care	7	20 (100)	Very high level of consensus/Very high priority	1
2.	Coordinated and joined up care	7	19 (95)	Very high level of consensus/Very high priority	2
3.	Flexible systems in place to support people (before ‘cliff-edge’ moments when their conditions or circumstances change)*ˆ	6	18 (90)	Very high level of consensus/Very high priority	3
4.	Support for mental health and emotional wellbeing^a^	6	17 (85)	High level of consensus/High priority	4
5.	Data sharing across care services and linked electronic health records	6	17 (85)	High level of consensus/High priority	4
6.	Training in multiple long term conditions for healthcare and social care professionals	6	17 (85)	High level of consensus/High priority	4
7.	Access to social care, community-based services and other provision	6	17 (85)	High level of consensus/High priority	4
8.	Thorough assessment of health and social care needs	6	17 (85)	High level of consensus/High priority	4
9.	Early intervention and preventative approach	6	17 (85)	High level of consensus/High priority	4
10.	Continuity of care	6	17 (85)	High level of consensus/High priority	4
11.	Service availability in terms of timeliness and geography	6	17 (85)	High level of consensus/High priority	4
12.	Supporting self-management of conditions	6	16 (80)	High level of consensus/High priority	5
13.	Symptom management and monitoring	6	15 (75)	Fairly high level of consensus below threshold	6
14.	Prescribing and medication management	6	14 (70)	Fairly level of consensus below threshold	7
15.	Inclusive/Equitable service provision	6	14 (70)	Fairly high level of consensus below threshold	7
16.	Support with daily living and independent living^a^	6	14 (70)	Fairly high level of consensus below threshold	7
17.	Addressing loneliness and social isolation	6	14 (70)	Fairly high level of consensus below threshold	7
18.	Finance/financial assistance	6	13 (65)	Some level of consensus below threshold	8
19.	Support provided to informal carers	6	13 (65)	Some level of consensus below threshold	8
20.	Access to information for patients and carers and where to go for support	6	12 (60)	Some level of consensus below threshold	9
21.	Environmental factors and wider social determinants of health	6	12 (60)	Some level of consensus below threshold	9
22.	Able to engage in meaningful activities and social participation^a^	6	12 (60)	Some level of consensus below threshold	9
23.	Recognition of and support with lifestyle factors	6	11 (55)	Some level of consensus below threshold	10
24.	Support for children of people living with multiple long term conditions^a^,^b^	6	11 (55)	Some level of consensus below threshold	10
25.	Housing/accommodation that meets individual's needs	6	11 (55)	Some level of consensus below threshold	10
26.	Work issues and training for people living with multiple long term conditions^a^	5	9 (45)	No consensus	11
27.	Enhanced support from family and other informal carers	5	8 (40)	No consensus	12
28.	Increasing knowledge and awareness of multiple long term conditions among patients, carers and people in the wider community^a^	5	8 (40)	No consensus	12
29.	Support network	5	7 (35)	No consensus	13
30.	More intervention from private care providers^a^,^b^	5	6 (30)	No consensus	14
31.	Use of technologies to support individuals at home	5	4 (20)	No consensus	15

Factors reached consensus threshold if ≥80% rated, either 6 or 7 on Likert Scale, are shown in **bold**.

^a^Factors not aligned with the SELFIE Framework.

^b^Additional determinants suggested by respondents in round 2.

Consensus was not reached in the other 19 determinants, although 13 of these achieved some degree of consensus, albeit below our pre-determined threshold. For the remaining six determinants, there was no consensus among respondents.

There were three areas of very high consensus. All participants (100%) agreed that ‘person-centred and holistic care’ was extremely important. Nineteen (95%) and 18 (90%) out of 20 participants rated ‘coordinated and joined-up care’ and ‘flexible systems in place to support people (before ‘cliff-edge’ moments when their conditions or circumstances change)’, as extremely important (Table 5).

Eight other determinants equally achieved a high level of consensus, with 17 out 20 (85%) of participants rating these as very important (1. Support for mental health and emotional well-being; 2. Data sharing across care services and linked electronic health records; 3. Training in multiple long term conditions for healthcare and social care professionals; 4. Access to social care, community-based services and other provision; 5. Thorough assessment of health and social care needs; 6. Early intervention and preventative approach; 7. Continuity of care; 8. Service availability in terms of timeliness and geography). One other determinant, supporting self-management of conditions reached the consensus threshold, with 16 out of 20 respondents (80%) rating this determinant as very important.

Comparing these results with round 2 rankings, it is evident that over each round of the study, ‘person-centred and holistic care’ and ‘coordinated and joined up care’ consistently achieved very high levels of consensus, indicating these were considered by respondents to be the highest priority determinants for improving care in multimorbidity. Further, in the final round of deliberation, the additional determinant suggested in round 2, ‘flexible systems in place to support people (before ‘cliff-edge’ moments when their conditions or circumstances change), emerged as the third highest rated determinant. Other determinants suggested in round 2 (‘more intervention from private care providers’ and ‘support for children of people living with multiple long term conditions’) did not achieve the consensus threshold. A notable change in ranking was observed in relation to the determinant ‘housing/accommodation that meets individual's needs’, which respondents rated in the top five of priority determinants in round 2 but failed to reach the consensus threshold in round 3. Apart from the examples discussed above, respondents in round 3 did not significantly change their previous rating of each determinant from round 2, indicating a level of consistency in the prioritisation responses throughout the study.

Of the eight respondents who left various comments in the free-text box, two mentioned how they changed their responses in round 3. One respondent changed a number of responses based on their reflections of the wider Delphi group results while another mentioned that their re-rating was not affected by the group results. Five of the eight respondents left comments, including: 1) all determinants were important and that they overlapped to some extent; 2) most of the determinants were significant for different people at different stages of their health trajectory.

### Multimorbidity priority determinants and the SELFIE Framework

The significant majority of multimorbidity determinants (i.e. 24 out of 31 or 77%) (Table 5) identified by respondents aligned conceptually with one or more of the SELFIE framework categories.^14^ Comparing these priority determinants to the six health systems components of the framework,^14^ the majority were weighted towards the ‘Service delivery’ component of the framework, and to a lesser extent the ‘Technologies and medical products’, ‘Workforce’ and ‘Financing’ components.

Service delivery responses focused on the need for tailored, personalised services including treatment interaction (between the patient and care providers), patient self-management, the need for multi-disciplinary teams and ‘named’ care coordinators, informal carer involvement in decisions made about care options and maintaining continuity of care. Additionally, there was an emphasis on policies ensuring that services were both available and accessible in local communities, especially for patients with restricted mobility or limited transport options.

Technologies and medical product responses prioritised the need for shared information and monitoring systems across organisations. These included technologies which enhance information sharing and access relevant to frontline practitioners and patients (especially in relation to self-management of conditions), such as electronic medical records, patient portals, e-health tools and assistive technologies.

Workforce responses focused on structures and coordination roles that facilitate integrated care across organisational boundaries, in particular multidisciplinary teams and a named care coordinator or case manager. Further, there was an emphasis on continuous professional development in multimorbidity for care professionals, and provision of informal caregiver support, a cohort who often play a critical role in the care process.^14^

In relation to the Financing component of the framework, aspects of financial support for those with multimorbidity were emphasised. These included: “coverage and reimbursement of the interventions included in person-centred integrated care programmes [which] need to be generous enough to ensure equity in financial access for those who need them”; and out-of-pocket costs, which “may influence access, [non]adherence, and how and which care is used”.^14^ In addition, financial incentives were prioritised which can be “used to motivate persons with multi-morbidity to participate in and adhere to integrated care programmes”,^14^ as well as the need for policymakers to ensure equity and equal access to services for people with multimorbidity, especially those from lower socio-economic backgrounds.

Relating the priority determinants to the SELFIE scales of integration, the majority were located at the micro scale (n=9) of the framework. This is the scale concerned with the frontline delivery of care and other support to the individual. This indicates a prioritisation among respondents focused on promoting integrated care for those living with multimorbidity, which is personalised to individual care needs across all components of the health system.

Five determinants aligned to the meso scale and two to the macro scale. The meso and macro scales in the SELFIE framework are concerned with the policy, planning, organisational and operational components that underpin effective delivery of integrated frontline care provision. The determinants prioritised by respondents mainly emphasised the need for coordinated services, processes and structures which facilitate joined-up care, in particular shared information systems, organisational integration, interoperable systems and specific training and coordinated professional development for care practitioners in multimorbidity.

Virtually all determinants aligned with the core of the SELFIE model (which in relation to the hierarchy of scales in the SELFIE model can be understood as the individual scale), that is the holistic understanding of an individual’s specific care needs and capabilities to sustain and manage their care. All of the environment elements at the core of the framework (which also encompass wider social determinants of health) aligned with the priority determinants identified by our study.

A number of priority determinants (7 of 31 or 22.5%) identified were not closely aligned with or did not feature prominently in the SELFIE framework, although some of these were considered by respondents to be relevant to established multimorbidity (Table 5). The determinant ‘support for mental health and emotional well-being’ consistently received a high ranking from participants throughout the study. Two other determinants, which did not reach the consensus threshold, were nevertheless, considered to be ‘very important’ in relation to improving care in multimorbidity by 70% of respondents (‘Support with daily living and independent living’) and 60% (‘Able to engage in meaningful activities and social participation’) respectively. Interestingly, these determinants highlight the importance of a holistic approach to care in multimorbidity encompassing an individual’s daily social care needs, addressing their psychological and mental well-being and wider social needs.

## Discussion

In this study, we used the Delphi method to elicit and prioritise determinants of improved care in multimorbidity. A hierarchy of the key priority determinants was identified by our diverse panel of patients, carers, health and social care professionals, data scientists, and researchers in multimorbidity. There was a near unanimity of consensus among respondents that the primary priority determinants of improved care in multimorbidity were ‘person-centred and holistic care’ (ranked 1) and ‘coordinated and joined up care’ (ranked 2). Further, the determinant ‘flexible systems in place to support people (before ‘cliff-edge’ moments when their conditions or circumstances change)’ (ranked 3), also achieved a very high level of consensus among the panel. These priority determinants suggest that improving care for those living with multimorbidity requires a paradigm change in the current model of care and practice. In other words, there is a need for a more concerted move towards a holistic and individualised care model, built around the needs of the patient, which is organised and delivered by integrated care services to ensure continuity of care and seamless transitions in care between service providers. Similar findings have also been reported by a range of other studies examining improved care in multimorbidity.^1,2,9,26–28^

Other priority determinants for improved care which achieved consensus reflected the complex and diverse range of medical and non-medical care needs associated with multimorbidity and issues relating to access to services. These included support for mental health and well-being for those with multimorbidity, access to and availability of social services and other provision in local communities and comprehensive assessment of health and social care needs of individuals with multimorbidity. Supporting patients with self-management of conditions was identified as a priority determinant, highlighting a need for a shift towards greater patient participation in their care, which is “an essential component of chronic disease management and secondary prevention”,^29^ as well as being an important dimension of the person-centred care agenda.

Whilst there was a strong focus on improved frontline care at the level of the individual, our findings also reflected an emphasis on macro factors relating to systems change and service reconfiguration. These included data sharing across care services, linked electronic health records, continuity of care and early intervention and preventative approaches,^26^ the latter also indicating a need to address upstream social and non-medical determinants that can significantly influence health trajectories and outcomes.^30^

An interesting finding from our study was the identification of training in multiple long term conditions for healthcare and social care professionals as a priority determinant. This is an issue which has received little attention in the literature^26^ and may indicate that current training programmes for healthcare and social care professionals continue to be specialist and designed around treatment and care for single conditions, and as such fail to provide care personnel with the requisite skills and knowledge that is specific to care for multimorbidity.

Overall, many of our principal findings are reflected in the wider literature^8^ and are indicative of a care system in the UK that is still predominantly structured and delivered on the basis of care for single conditions, with care practice and organisational cultures also similarly orientated. In such an institutional and operational form, the system struggles to address holistically, the multifaceted and complex care needs associated with multimorbidity.^2^ Considered from a strategic policy perspective, the key priority determinants identified may be a recognition that efforts to advance person-centred care, which is holistic, integrated and addresses a range of interrelated medical and non-medical care needs of multimorbidity patients, continues to be slow, and implementation remains a major policy and practice challenge both in the UK and internationally.^2,4,8,27^ An important learning point for policy-makers from our study is that these are likely to continue to feature as critical determinants influencing care in multimorbidity until further significant progress is made towards redesigning care services and delivery around the needs of the individual rather than service providers.^26,27^ In relation to care practice, our findings suggest that clinicians and care practitioners need to take further steps to ensure wider non-medical determinants and care needs are fully assessed and taken into consideration in care plans and subsequent delivery of care to those with multimorbidity.^2,10^

## Strengths and limitations

Based on our knowledge of the literature, this is one of the first studies to use the Delphi method to identify and prioritise those medical and non-medical determinants that are key to improving the care of people with multimorbidity. The main strength of our study is the diverse and balanced panel of respondents spread across the age range, including people living with multiple conditions and those providing informal and formal care. This provided a more comprehensive understanding of multimorbidity and determinants of importance. The online Delphi approach may have contributed to capturing a geographically diverse UK-wide sample, as well as contributing to the high response rate (83%), which is above most recommendations.^31,32^ Three rounds were deemed to be the appropriate duration, as the results obtained in the second and third rounds did not change significantly, indicating it was unlikely important additional data would have been generated by holding further rounds of deliberation. Extending the duration of the study may also have affected the response rate, potentially undermining the validity of the results.^24^

However, as an online Delphi study, the absence of an interactive in-person discussion may have limited the richness of data collection as respondents were unable to engage in dialogue and exchange opinions with others on the panel regarding prioritisation of determinants. While our sample size was within the accepted range for Delphi studies,^21^ a relatively low number of respondents participated in the study (n=20), the significant majority of whom (83%) were White British, which may have limited the range of perspectives and diversity of experiences captured. Further, a greater number of study participants may have enabled the collection of a broader range of perspectives on the topic area and produced more data. We do not know why only 32 of the 56 respondents who initially expressed an interest in participating in the study, subsequently decided not to do so. Nevertheless, it is important to note that our sample remained stable throughout, with little respondent ‘drop-out’ recorded, a factor which may have supported a consistency of approach in reaching a final consensus of opinion.

Ranking of the determinants is inherently a subjective process influenced by respondents’ lived experience, specific knowledge and expertise, as well as professional backgrounds and training, etc. In this respect, the majority of our study participants were care professionals or from other professional backgrounds, sample characteristics which may have biased the selection and weighting of priority determinants.

Additionally, as some respondents commented, it can be difficult to assign a weighting to individual determinants, as prioritisation can be dependent on a complex range of variables and risks such as a patient’s specific personal circumstances including socioeconomic status, the environmental and geographical context in which they are situated, as well as the stage in their disease trajectory, etc. Prioritisation is also a challenge because determinants are often inextricably interrelated and interdependent, making it difficult to separate out and identify a hierarchy of importance in relation to improving care in multimorbidity.

## Conclusion

Our study highlights the complex range of interrelated medical and non-medical determinants influencing care in multimorbidity. Current understanding of and approaches to care are still too focused on the medical dimensions of care and priorities of systems and service providers.^26^ However, the care needs of those living with multiple health conditions are often broader, encompassing biopsychosocial needs (e.g. mental health and emotional wellbeing)^2,26^ and social circumstances including the impact on health and well-being of the wider environment in which an individual is situated.^10^ Our study supports the growing body of evidence that further progress towards a holistic and person-centred care model^2,26,33^ is urgently required, which empowers people to be full and equal participants in their care decision-making to ensure that care delivery can be further optimised to address the diverse range of individual care needs often associated with multimorbidity.^2,26,34^ This requires a cultural shift in contemporary healthcare and social care philosophy and practice,^26^ necessitating a move ‘from a single disease-focused biomedical model to a biopsychosocial’ approach,^12^ as well as further progress towards an integrated care system.^2^ While some progress has been made in this direction in recent years,^2^ such a shift in approach may also need to be complemented by a greater emphasis and re-orientation towards preventative upstream approaches.^35^ This will necessitate directing increased expenditure on preventative public health activities including support to those agencies (e.g. local government) responsible for social care and addressing wider social or non-medical determinants of health.^30^ A recent study^6^ highlighted the importance of targeting resources towards preventative activities in relation to multimorbidity, concluding that: ‘multimorbidity appears to be associated with per capita public health expenditure, and health-related quality of life with social care expenditure.’

Additionally, the implementation of new care governance and delivery models such as Integrated Care Systems (ICS) in England may also provide a mechanism for the integrated delivery of tailored person-centred care.^36^ ICS are partnership bodies consisting of care provider organisations and local authorities, the purpose of which is to plan and deliver joined-up health and care services.^37^ Whilst there are tentative signs that ICS may have the potential to enhance care integration,^37^ the concept is a recent innovation and as a result, it is too early to make an assessment as to whether this model will be able to deliver the integrated and personalised models of care which policymakers envisage.

## Supplemental Material

Supplemental Material - Eliciting and prioritising determinants of improved care in multimorbidity: A modified online Delphi study Click here for additional data file. Supplemental Material for Eliciting and prioritising determinants of improved care in multimorbidity: A modified online Delphi study by Glenn Simpson, Beth Stuart, Marisza Hijryana, Ralph Kwame Akyea, Jonathan Stokes, Jon Gibson, Karen Jones, Leanne Morrison, Miriam Santer, Michael Boniface, Zlatko Zlatev, Andrew Farmer and Hajira Dambha-Miller in Journal of Multimorbidity and Comorbidity

## Ethical Statement

### Ethics approval

Ethical approval for the study was provided by the Faculty of Medicine Ethics Committee, University Hospital Southampton (reference number 67953).

### Patient Consent

Informed written consent was obtained from all participants.
