# Corrigendum to “Eliciting and prioritising determinants of improved care in multimorbidity: A modified online Delphi study”

**DOI:** 10.1177/26335565241279342

**Published:** 2024-10-03

Simpson G, Stuart B, Hijryana M, et al. Eliciting and prioritising determinants of improved care in multimorbidity: A modified online Delphi study. *J Multimorb Comorb* 2023;13. doi:10.1177/26335565231194552

In this article, the following information has been added to the Funding Section: AF was supported by the National Institute of Health Research (NIHR) Oxford Biomedical Research Centre (BRC). The corrected funding statement is provided below:

The Primary Care Research Centre at the University of Southampton is a member of the NIHR School for Primary Care Research and is supported by NIHR Research funds. HDM is a National Institute for Health Research funded Academic Clinical Lecturer and has received NIHR funding for this grant (NIHR202637). This report is independent research funded by the National Institute for Health Research (Artificial Intelligence for Multiple Long Term Conditions (AIM), “The development and validation of population clusters for integrating health and social care: A mixed-methods study on Multiple Long Term Conditions”, “NIHR202637”). The views expressed in this publication are those of the author(s) and not necessarily those of the NHS, the National Institute for Health Research or the Department of Health and Social Care. AF was supported by the National Institute of Health Research (NIHR) Oxford Biomedical Research Centre (BRC).

